# Weather extremes and perinatal mortality – Seasonal and ethnic differences in northern Sweden, 1800-1895

**DOI:** 10.1371/journal.pone.0223538

**Published:** 2019-10-22

**Authors:** Barbara Schumann, Erling Häggström Lundevaller, Lena Karlsson

**Affiliations:** 1 Centre for Demographic and Ageing Research (CEDAR), Umeå University, Umeå, Sweden; 2 Department of Epidemiology and Global Health, Umeå University, Umeå, Sweden; 3 Department of Sociology, Umeå University, Umeå, Sweden; Columbia University, UNITED STATES

## Abstract

**Background:**

Many studies have shown the impact of heat and cold on total and age-specific mortality, but knowledge gaps remain regarding weather vulnerability of very young infants. This study assessed the association of temperature extremes with perinatal mortality (stillbirths and deaths in the first week of life), among two ethnic groups in pre-industrial northern Sweden.

**Methods:**

We used population data of indigenous Sami and non-Sami in selected parishes of northern Sweden, 1800–1895, and monthly temperature data. Multiple logistic regression models were conducted to estimate the association of cold (<10^th^ percentile of temperature) and warmth (>90^th^ percentile) in the month of birth with perinatal mortality, adjusted for cold and warmth in the month prior birth and period, stratified by season and ethnicity.

**Results:**

Perinatal mortality was slightly higher in Sami than in non-Sami (46 vs. 42 / 1000 live and stillbirths), but showed large variations across the region and over time. Both groups saw the highest perinatal mortality in autumn. For Sami, winter was a high-risk time as well, while for non-Sami, seasonality was less distinct. We found an association between exposure to cold and perinatal mortality among winter-born Sami [Odds ratio (OR) 1.91, 95% confidence interval (CI) 1.26–2.92, compared to moderate temperature], while there was little effect of cold or warmth during other seasons. Non-Sami, meanwhile, were affected in summer by warmth (OR 0.20, CI 0.05–0.81), and in autumn by cold (OR 0.39, CI 0.19–0.82).

**Conclusions:**

In this pre-industrial, subarctic setting, the indigenous Sami’s perinatal mortality was influenced by extreme cold in winter, while non-Sami seemed to benefit from high temperature in summer and low temperature in autumn. Climate vulnerability of these two ethnic groups sharing the same environment was shaped by their specific lifestyles and living conditions.

## Introduction

A large number of studies have shown seasonal variations of mortality, as well as negative effects of heat and cold on human health. Climate vulnerability–the risk to be negatively affected by weather–depends on individual characteristics, such as biological susceptibility, age, gender, premorbidity, behaviour and socio-economic status. Even contextual factors such as local environment and climate, building material and social structure can exacerbate temperature-related risk [1–5]. Children and infants have been shown to be particularly vulnerable to temperature extremes [6, 7], although research inconsistencies and knowledge gaps remain [8].

Generally, mortality among infants is highest during the first four weeks of life (neonatal mortality), and decreases afterwards [9]. An estimated 75% of all neonatal deaths occur in the first week (early neonatal mortality) [10]. Perinatal mortality is the rate of stillbirths and early neonatal mortality; rates are reported as cases per 1000 still- and live births [11]. In today’s global statistics, about half of all perinatal deaths are stillbirths, of which one third is caused by complications during birth and could be prevented [12]. Preterm birth, low birth weight and stillbirth are birth outcomes related to perinatal mortality; their determinants are genetic, socio-economic, environmental and behavioural factors [13]. Intranatal complications, congenital defects and preterm birth are the main causes of early neonatal death [14], but also the mother’s age, health status and mortality during birth. Thus most early deaths could be prevented, although prevention of perinatal mortality is considered more difficult than of infant mortality [11].

There is a lack of research into the role of meteorological factors for neonatal mortality (e.g. [14]; focus of the published studies is on heat exposure during pregnancy as a risk factor for adverse birth outcomes in contemporary societies [13, 15]. Little is known about the vulnerability of newborns to extreme weather conditions in subarctic settings prior to urbanization, economic development and industrialization. A better understanding of environmental risks such as heat and cold extremes, in particular for perinatal deaths in such resource-poor societies, could contribute to preventing these deaths.

A number of studies have addressed seasonality and heat and cold effects on infant mortality (death in the first 12 months) in historical and modern societies, but results are highly divergent. In 19^th^ century Italy, being born in winter posed a higher risk for infants, compared to summer-born infants, while in colder regions such as Russia or Poland, summer, but not winter was a high risk [16, 17]. On the other hand, a study comparing contemporary perinatal mortality rates across Arctic and subarctic locations showed that average temperature in January was protective: The milder the local winter climate, the lower the perinatal death rates. This relationship remained even if adjusted for socio-economic factors that might vary across locations [18]. How local temporal variations of winter temperature were associated with perinatal mortality has however not been investigated in this cross-country study. In countries with a cold climate, adverse birth outcomes (stillbirth, low birth weights and preterm birth) are reportedly more prevalent in winter than in other seasons [13]. Also birth rates have been studied in relationship to seasonality; however, the role of temperature, other climatological factors and seasonal patterns of human activities contains still many open questions [13, 19, 20].

### Causal mechanisms of temperature-perinatal mortality relationships

Different causal mechanisms have been suggested for adverse effects of high and low temperatures on birth outcomes and early neonatal deaths. Heat exposure might lead to stillbirth, because the mother, due to a higher fat deposition and weight gain during pregnancy, is less able to cope with heat stress, for example through sweating. This might cause body dehydration, cell damage and insufficient foetal nutrition, and increase the risk of preterm births or stillbirths [13, 21].

Infants are highly susceptible to both heat and cold exposure because they lack the ability for efficient thermoregulation, and have a higher metabolic rate and temperature transfer between the environment and the body than adults. They therefore are at high risk of heat stress [8, 13, 21]. Also hypothermia, associated with mortality, is common among neonates [10]. Even in tropical countries, low ambient temperatures pose a threat for the unborn and newborn due to hypothermia; cold-related risk is highly dependent on living conditions [10, 22]. During pregnancy, cold exposure (often associated with indoor air pollution from biomass burning) might affect foetal growth negatively, increasing the risk for stillbirth or preterm birth [13, 21, 23].

Climate vulnerability might differ between early neonatal and late neonatal children. In the mid-1800s Veneto region in Italy, mortality was highest in winter-born infants due to excess deaths in the first week [24]; in Venice, a large effect of low temperature was found for early neonatal mortality, but less profound for late neonatal mortality [22]. Studies have shown that preterm birth, related to poor living standards, maternal malnutrition and low birth weight, increases the risk of hypothermia and early neonatal mortality [25], while respiratory diseases are a common cause of death in the second and third week of life [24].

### Infant mortality in pre-industrial Sweden

In pre- and industrializing societies and in today’s low- and middle-income countries, perinatal death rates are considerably higher than in modern high-income countries [11, 12]. In Sweden, rates have been declining considerably since the 19^th^ century. In the rural towns of Skellefteå and Sundsvall, perinatal mortality in 1830–1899 was around 40/1000; rates were highest in the 1860s-70s, following similar trends of infant, maternal and total mortality. Perinatal rates fell at the end of the century, mainly due to the increased presence of midwives [11].

Northern Sweden–Sápmi, covering two third of the whole country–has been the home of the indigenous Sami people for centuries. Historically, their main economy was based on reindeer herding but also from hunting and fishing. Over time, other ethnic groups (hereafter named “non-Sami”) migrated from other regions to Sápmi to work with small-scale agriculture, as lumberjacks or–e.g. in Gällivare parish–as miners [26, 27]. At the beginning of the 19^th^ century, Sami were the majority in northern Sweden, but became a minority until the end of the century [27].

Nineteenth century Sápmi provides an interesting environmental health perspective: This period marked in Sweden the end of the “little ice age”; the early 1800s were one of the coldest periods of the last 1000 years; also the 1860s saw very low temperatures [28]. It was a pre-industrial setting, inhabited by two ethnic groups–Sami and non-Sami (Swedes and Finns)–who shared the same environment and climate, but differed in lifestyle, economic and cultural practices, and in the extent of multigenerational adaptation to harsh subarctic living conditions. The Sami reindeer herders lived often as semi-nomads, following the seasonal routes of their herds, and spent most time outdoors. In winter, they were exposed to very low ambient temperatures, while the non-Sami farming communities lived in houses better protected from the cold, and had less work in winter. On the other hand farmers, including pregnant women had in summer the highest work load, in particular during harvest time [29].

In 1800s Sweden, mortality was high, although there were substantial regional differences, with those living in southern Sápmi enjoying higher life expectancies than the rest of the country [26]. In the province of Norrbotten, covering the northern part of Sápmi, infant mortality was 136 per 1000 live births in the late 1800s, while it was only 92/1000 in Jämtland (southern Sápmi) [30]. Total mortality was reportedly higher for Sami than for non-Sami, except for the older age groups. In some Sápmi regions, infant mortality rates were twice as high for Sami as for non-Sami [26, 27]. During the late 1800s, however, mortality rates of the two ethnic groups approached each other [26].

We have previously reported seasonal patterns of neonatal mortality in northern Sweden prior to industrialization, but also ethnical differences in these patterns [20]. While Sami had the lowest neonatal mortality during summer, no effect of seasonality was observed for non-Sami. Regarding early neonatal mortality, autumn-born appeared least vulnerable among Sami, and most vulnerable among non-Sami, compared to winter-born children, although differences were statistically non-significant [29]. Stillbirth rates in the 19^th^ century were for Sami lowest in summer and highest in autumn, while we found very little seasonality in non-Sami [20].

We also found that neonatal mortality rates were related to cold weather in winter among Sami, but not among non-Sami. Neither group was vulnerable to warm extremes in summer [31]. Our studies from Skellefteå, a rural town in northern Sweden, furthermore indicated an overall protective effect of higher temperature [32], but also that the impact of weather on total mortality depended on the season [33, 34], and varied by age, with the youngest ones (under three years of age) being least vulnerable to low temperature [34].

For the present study, we hypothesized that the role of temperature extremes for perinatal mortality depended a) on the season of the year and b) on ethnicity. The season might modify weather impacts, as warmth during winter is likely to prevent hypothermia and cold-related infections, while in summer it might increase mortality due to heat stress of the pregnant mother or newborn. Furthermore, we assumed that climate vulnerability is to a large degree modified by living conditions and behaviours, related to socio-economic and cultural factors. Ethnicity determined the seasonal activities of the mother–non-Sami farmers being on the field in summer, but mostly inside in winter, while Sami were more exposed to harsh outdoor climates in winter. As such, ethnicity in this historical setting can be seen as an indicator for socio-economic and cultural-behavioural differences between Sami and non-Sami.

Therefore, this study aimed at investigating the association of warm and cold weather with perinatal mortality among Sami and non-Sami in Swedish Sápmi during the 19^th^ century.

## Material and methods

### Study population

**Fig 1:**
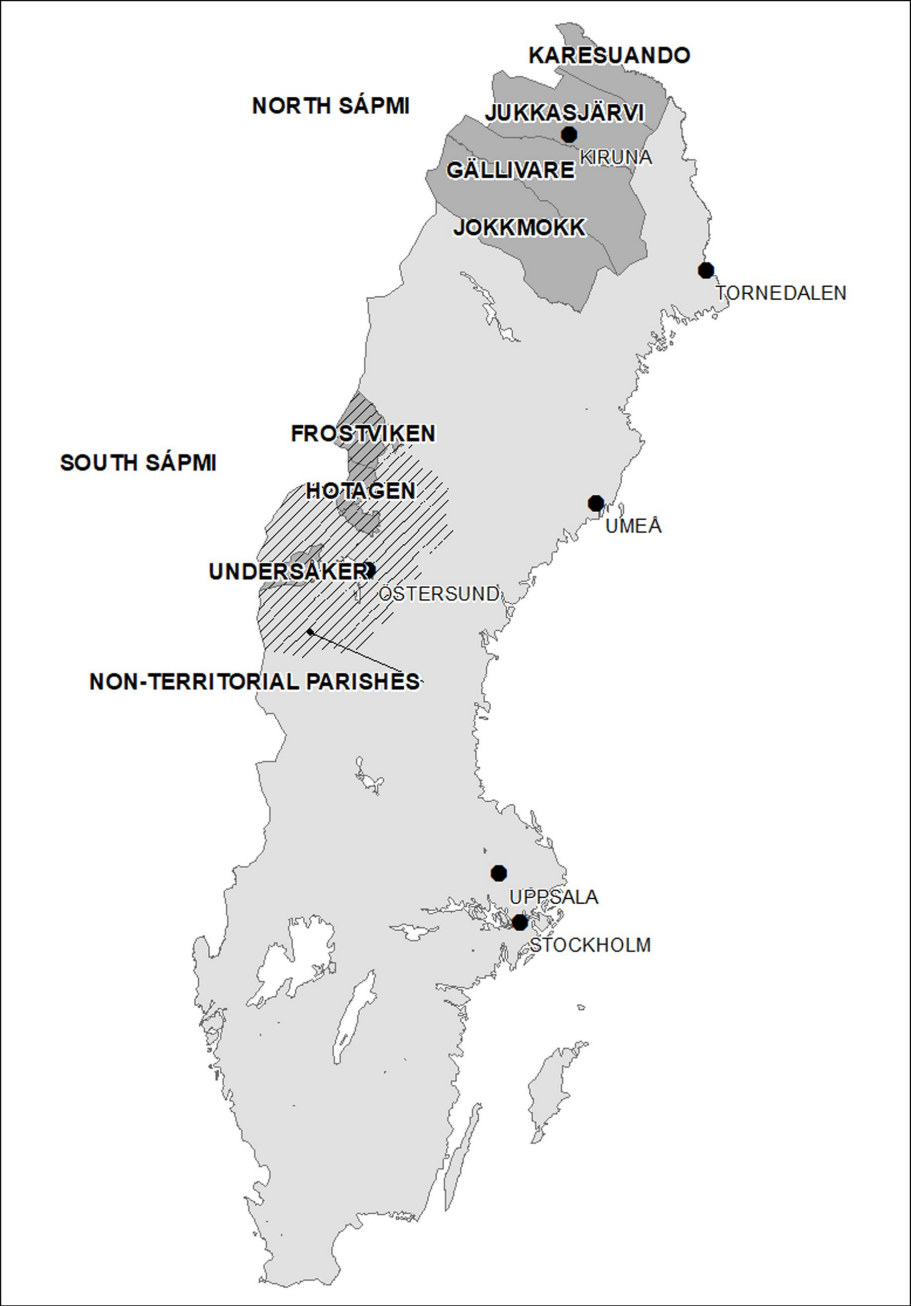
Map of Sweden, including parishes in the study area of Swedish Sápmi 1800–1895. Source: Swedish National Archives (Riksarkivet), modified by Demographic Data Base, Umeå University (added locations of weather stations). Original map licenced under CC0.

The study was based on population registers (ethical clearance from the regional ethical committee in Umeå (EPN); no.2017/509-31). Data were obtained from the Demographic Database, Umeå University, for the period of 1800 to 1895. Vital data were reported in ecclesiastic parish registers; in southern Sápmi, the Sami were usually allocated to administrative, non-territorial entity Sami parishes [26, 35]. Our study region includes parishes of northern Sápmi (Jokkmokk, Gällivare, Jukkasjärvi, and Karesuando, in today´s Norrbotten county), hereafter named “north Sápmi”, and southern Sápmi (parishes of Undersåker, Föllinge, Hotagen, Frostviken, and Hede, today’s Jämtland county), hereafter “south Sápmi” (Fig 1). The region is a hilly or mountainous terrain with boreal forest, in the north also with tundra landscape.

Parish ministers noted the date and place of birth, whether the child was born alive or dead, date and place of death, sex and a number of family-related details. It was up to the minister to state if it was a still- or life birth; there might have been misclassifications regarding the birth outcome and the exact date of death. While the Swedish quality of registers, in particular of early deaths, was not perfect, it was considered rather accurate in the second half of the 19^th^ century [36].

Ethnicity was not recorded by ministers, but could be determined indirectly based on ethnic markers referring to Sami in the registries. These markers include parental occupation (e.g. reindeer owner–the predominant occupation of Sami, but not other Swedes at that time), the terms “Lapp” or “nomad” (used at that time exclusively for Sami) or typical family names used only by Sami. Geographical location of parishes also indicated that these were inhabited exclusively or predominantly by Sami. An indicator of ethnicity based on these entries by the parish minister has previously been developed [37] and has been used in a number of studies to distinguish between ethnic Sami and non-Sami living in Sápmi prior to 1900. Details of the categorization into these two groups can be found in [27].

The dataset used in this study contains information about births, deaths, and other vital data of residents in the selected parishes. We included all children born (either still- or live births) between 1800 and 1895, with reported place of birth, date of birth, place and date of death, and ethnicity (Sami or non-Sami). Children who were born outside the study region were excluded. Perinatal death, the outcome variable in this study, was defined as stillbirth or death in the first week of life (day 0–6). Definitions of stillbirth changed over time [36], and a number of stillbirths might have been recorded as infants born alive who died on the first day of live, and vice versa, impeding a differentiation of stillbirths and early neonatal deaths. Therefore, the two groups were combined as perinatal deaths in our analyses.

### Temperature data

We used temperature data from Tornedalen in north Sápmi (monthly means 1802–1895, homogenized and validated by [38], and reconstructed temperature data for Umeå in south Sápmi (monthly means 1800–1895). Umeå data were estimated based on measurements from Tornedalen and Uppsala (details of the reconstruction in [34]). Thus, temperature series were available for the north and for the south of the study region, either as homogenized or as reconstructed data. These weather data therefore allow analyses only on a monthly, not a daily scale. They also have the limitation of being obtained from weather stations outside the study region (Fig 1).

The main exposure variables were categorized as cold and warm extremes in the month of birth, based on the mean temperature of each calendar month. Cold extremes were defined as temperature below the 10^th^ percentile, and warm extremes as temperature above the 90^th^ percentile of each month. Most contemporary studies use cut-offs at the 5^th^ and 95^th^ percentile, respectively, whereas some choose the 20^th^ and 80^th^ percentiles. We decided however for an approach based on the 10^th^ and 90^th^ percentiles for cold and warm extremes in order to obtain an adequate number of exposed cases, while differentiating sufficiently between moderate temperature and extremes at the high and the low end. Each calendar month was thus classified as either cold, warm or moderate. Furthermore, exposure to temperature extremes in the month prior birth (lag 1) was considered as a co-variable. For children born in north Sápmi, Tornedalen data were used, and for children born in south Sápmi, Umeå data were used.

Months of birth were grouped by season (December to February as winter, March to May as spring, June to August as summer, and September to November as autumn).

### Statistical methods

The final dataset contained individual information about birth and death date, ethnicity, mortality (perinatal death), sex, period of birth (1800–1829, 1830–1859 or 1860–1895), region (north or south Sápmi), temperature (cold, moderate or warm) at birth and at lag 1 month, for each infant born alive or dead during the study period 1800–1895. Perinatal deaths in Sami and non-Sami were presented as absolute numbers and rates per thousand for all births, and by region, period, season of birth, and temperature extremes at birth.

The association of temperature extremes at birth and perinatal mortality was modelled by logistic regression, stratified by season and ethnicity. Model 1 included only warm and cold extremes in the month of birth, model 2 added a lagged effect (lag 1—warm and cold extremes in the month prior birth). The main model 3 adjusted for period and temperature extremes at lag 1. An additional model 4 adjusted even for sex and region, in order to account for sex differences in mortality and for the fact that Sami and non-Sami were not equally represented in the north and the south, while mortality was on average higher in the north [27]. Odds ratios (OR) with 95% confidence intervals (CI) were computed, with moderate temperature (10^th^ to 90^th^ percentile) as the reference category. For the main model 3, we investigated whether effects of temperature extremes at birth differed by ethnicity by computing season-specific ratios of odds ratios (ROR) with 95% confidence intervals for Sami vs. non-Sami, based on the method by Altman and Bland [39]. Since the homogenized temperature data from Tornedalen (north Sápmi) were more reliable than the partly reconstructed data from Umeå (south Sápmi), we conducted sensitivity analyses for model 3 using only north Sápmi data.

The software R version 3.4.3, including the function *glm*, was used for all analyses.

## Results

### Descriptives

Among the Sami population, a total of 14,045 still- and live births was eligible for inclusion, while among non-Sami, the respective number was 14,194 births. Of these, 645 Sami (46‰) and 601 non-Sami (42‰) were born dead or died within the first week. Among Sami, 50.5% of all perinatal deaths were stillbirths; among non-Sami, the proportion was 51.9%. Table 1 summarizes perinatal mortality data during the study period. Overall, rates were higher among Sami than non-Sami; in both groups, deaths were more common in the northern regions of Sápmi than in the south. Perinatal mortality was highest in the beginning of the study period, declined in the middle of the 19^th^ century, and increased again after 1860. Seasonality appeared less distinct for non-Sami (perinatal mortality ranging from 40 to 44‰) than for Sami (42 to 52‰). Mortality was highest for autumn-born infants of either ethnicity (Sami 52‰, non-Sami 44‰). Sami infants were most at risk during unusually cold months, while there was little difference between moderate and very warm months. In non-Sami, on the other hand, mortality was lower during warm months, and no increase during cold months compared to moderate months was observed.

**Table 1:** Births (live and stillbirths) and perinatal deaths in Sami and non-Sami, 1800–1895.

	Sami		Non-Sami	
	Births incl. stillbirths N	Perinatal deaths N (‰ of all births)	Births incl. stillbirths N	Perinatal deaths N (‰ of all births)
**Total**	14,045	645 (46)	14,194	601 (42)
**Sex**				
**Male**	7,119	370 (52)	7,253	328 (45)
**Female**	6,926	275 (40)	6,941	273 (39)
**Region**				
**South**	1,885	66 (35)	5,054	162 (32)
**North**	12,160	579 (48)	9,140	439 (48)
**Period**				
**1800–1829**	2,915	184 (63)	1,355	58 (43)
**1830–1859**	4,296	130 (30)	2,901	76 (26)
**1860–1895**	6,834	331 (48)	9,938	467 (47)
**Season**				
**Winter**	3,905	183 (47)	3,891	157 (40)
**Spring**	3,716	156 (42)	3,723	159 (43)
**Summer**	2,989	126 (42)	3,349	142 (42)
**Autumn**	3,435	180 (52)	3,231	143 (44)
Temperature*				
**Cold**	1,453	74 (51)	1,456	61 (42)
**Moderate**	11,232	513 (46)	11,436	496 (43)
**Warm**	1,360	58 (43)	1,302	44 (34)

* Temperature: Cold <10^th^ percentile, moderate = 10-90^th^ percentile, warm > 90^th^ percentile of month-specific temperature;

Perinatal mortality: stillbirth or death in the first 7 days of life. Rates per thousand (‰) are for all births in each category.

Table 2 shows the cut-off temperature values for extreme cold and warm months in northern and southern Sápmi, exemplarily for one month of each season. In January, there was a difference of about 10°C between the 10^th^ and the 90^th^ percentile of monthly mean temperature, while the difference was less than 4°C in July.

**Table 2:** Temperature extremes, Umeå and Tornedalen, 1800–1895.

Monthly mean temperature, °C	Umeå	Tornedalen
**January**		
10^th^ percentile	-14.4	-18.6
Median	-8.7	-12.0
90^th^ percentile	-4.6	-7.5
**April**		
10^th^ percentile	-2.5	-4.7
Median	0.2	-1.5
90^th^ percentile	1.9	0.7
**July**		
10^th^ percentile	13.3	13.2
Median	14.8	14.8
90^th^ percentile	16.8	16.9
**October**		
10^th^ percentile	0.2	-1.4
Median	2.6	1.2
90^th^ percentile	5.2	4.4

Percentiles and median values were computed for each calendar month separately. The table shows the threshold values for one month of each season in Umeå and Tornedalen, respectively.

### Association of temperature extremes and perinatal mortality

**Fig 2:**
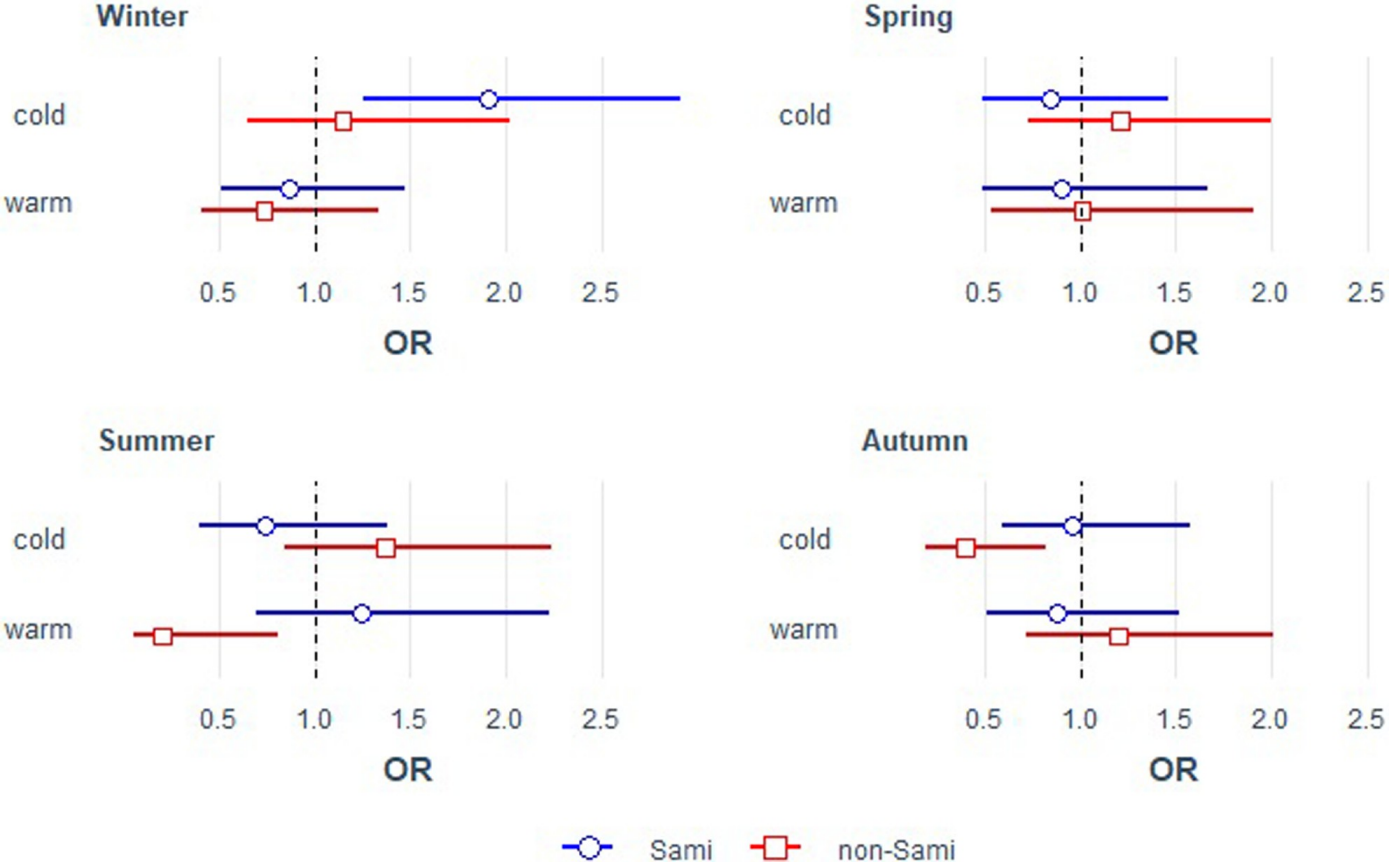
Perinatal mortality by temperature extremes at birth, stratified by season and ethnicity. Estimates are odds ratios (OR) with 95% confidence intervals for warm and cold extremes in the month of birth, adjusted for temperature extremes at lag 1 month and period (model 3).

Fig 2 shows results of model 3 for perinatal mortality by temperature extremes at birth, adjusted for temperature extremes at lag 1 month and period. Estimates for all included parameters of simple and multiple regression models (model 1 to 4) are provided in the supplement (S1 Table).

For winter-born Sami infants, perinatal mortality was almost twice as high during extreme cold months, compared to months with moderate temperatures (OR 1.91, CI 1.26–2.92). No such effect was seen for non-Sami. Warm months in winter were not associated with mortality in either ethnic group, and there was no relation of extreme temperature with mortality during spring.

In summer, cold appeared to increase the risk among non-Sami, although the confidence interval was large and included the reference point of 1 (OR 1.37, CI 0.84–2.24). Warm extremes, on the other hand, decreased perinatal mortality considerably (OR 0.20, CI 0.05–0.81). No effects were observed for summer-born Sami. Cold during autumn profoundly decreased the risk of perinatal death among non-Sami (OR 0.39, CI 0.19–0.82). Temperature extremes were not associated with perinatal mortality of Sami born in autumn.

In model 1 (considering only temperature extremes at birth), effects of warm and cold extremes at birth were generally slightly smaller, thus, controlling for lagged temperature and period (model 2, 3 and 4) amplified the effect. Model 4 (including also region and sex) showed higher perinatal mortality in the north and for boys, but adjustment for these factors did not change substantially the estimates for cold and warm effects (S1 Table).

When comparing the two groups’ season-specific estimates, we found some indication that cold-related risk in winter was larger for Sami than for non-Sami (ROR 1.67, 95% CI 0.82–3.39), and in autumn was substantially larger for Sami (ROR 2.43, CI 1.00–5.90) (Table 3). In summer, perinatal mortality risk due to warm extremes was more than six times higher for Sami than for non-Sami (ROR 6.27, CI 1.36–28.87), mainly due to the large protective effect of warmth on non-Sami mortality. Cold-related risk in summer appeared smaller in Sami than non-Sami (ROR 0.53, CI 0.24–1.19).

**Table 3:** Interaction of ethnicity and seasonal temperature extremes. Ratios with 95% confidence intervals of odds ratios for perinatal mortality, Sami vs. non-Sami.

	Winter	Spring	Summer	Autumn
**Cold**	1.67 (0.82–3.39)	0.69 (0.33–1.47)	0.53 (0.24–1.19)	2.43 (1.00–5.90)
**Warm**	1.18 (0.53–2.62)	0.89 (0.37–2.18)	6.27 (1.36–28.87)	0.73 (0.34–1.56)

Ratio of odds ratio (ROR) for Sami compared to non-Sami, per season, model 3: adjusted for cold and warm extremes at lag 1 and period of birth.

In sensitivity analyses including only births from north Sápmi and the more reliable homogenized temperature data from Tornedalen, effects of model 3, adjusting for lagged temperature extremes and period, were generally similar as for the total region. Among non-Sami, in particular cold effects were higher, while for Sami, estimates were slightly diminished (S2 Table).

## Discussion

In this study, we assessed the association between seasonal temperature extremes and perinatal mortality among two ethnic groups in northern Sweden before the onset of industrialization. These two groups–Sami reindeer herders and non-Sami settlers–shared the same environment and weather conditions, but differed regarding culture, socio-economic factors and living conditions.

During the 19^th^ century, total mortality decreased substantially in Sami, but not in non-Sami [27]. According to our data, perinatal mortality first declined and then increased in later decades, probably owing to better registration routines of deaths [36], but also to the presence of midwives [11]. At the end of the study period, Sami and non-Sami rates had approximated each other at around 48 ‰ perinatal deaths–similar to the rates in the rural towns of Skellefteå and Sundsvall [11]. These perinatal rates were, however, lower and less variable than those of the province of Bologna, Italy, in the late 19^th^ century, reportedly 50 to 150 ‰ [40].

For both Sami and non-Sami infants, perinatal mortality was highest in autumn. Apart from that, seasonality patterns differed between the two groups and were less distinct in non-Sami; winter posed a higher risk for Sami than for non-Sami. While temperature variations across seasons might partly explain seasonality effects on perinatal mortality, other season-related factors might be relevant as well, such as cultural practices and economic determinants of the mother’s health influencing her nutritional status.

Our results indicated that winter-born Sami were highly vulnerable to cold, with perinatal mortality almost doubling compared to moderate temperatures, while higher temperatures did not clearly lead to increased survival chances. Infants of non-Sami settlers, on the other hand, were less affected by temperature. This is in line with our previous study showing cold-related risks for neonatal mortality in Sami, but not in non-Sami [31]. In a Dutch population sample encompassing the years 1855 to 2006, cold impacts on infant mortality appeared inconsistent, indicating a certain adaptation to winter climates in this temperate region [41].

One might conclude that Sami, who over centuries have been adapted to the harsh Nordic climate, could cope fairly well with temperature variations, except for the very low extremes during winter, when risk of perinatal death was already high. Exposure to indoor pollution (burning of biomass for heating), a risk factor for early neonatal mortality [42], might have acted as a confounder or effect amplifier of low temperature. In the Italian Veneto region during the 19^th^ century, winter-born neonates were highly vulnerable to low temperature. Presumably socio-economic conditions at that time were extremely precarious, leading to deteriorating health of women, who would then give birth to weak infants with low survival chances. Cold-related mortality of these infants was highest during the first week of life [24]; thus, living conditions contributed to heightened climate-vulnerability of neonates.

For neither Sami nor non-Sami born in spring, there was an association of weather with perinatal mortality. In a previous study, we had shown a protective effect of high spring time temperature on total mortality, but no effect on children aged 0–2 in the rural town of Skellefteå [34]. Thus it appears that very young children were less sensitive to temperature variations during spring than during other seasons.

Among summer-born non-Sami, warm weather was protective and cold weather appeared to be a risk factor. For Sami infants, on the other hand, temperature effects were in opposite directions, but estimate precision was too low to allow for conclusions. Thus, also for summer warmth, ethnic differences were distinct, resulting in a six times higher odds ratio (ROR) among Sami infants compared to non-Sami. For our non-Sami population, summer was a time of high agricultural work load with potential neglect of infants [19, 29]. Given the overall colder climate in this subarctic setting, warmer weather in summer might have decreased the risk of hypothermia in newborns, leading to the observed decrease in perinatal mortality from low to high temperature.

Sami perinatal mortality rates were highest during autumn; we found, however, no effect of temperature extremes in this season. Vulnerability of autumn-born Sami was thus mostly related to seasonal practices of reindeer herding families rather than to weather conditions. This was the time when Sami were on the move with their herds to the winter settlements [43], thus a challenging period for mothers and their newborns. Surprisingly, for non-Sami, cold weather posed a rather strong protective factor in autumn, in line with findings from Skellefteå (inhabited mainly by non-Sami farmers at that time) [34]. One explanation is that low temperatures brought about an early end of the harvest work and allowed women and infants to spend more time indoors.

Summing up, in this pre-industrial, subarctic setting, Sami newborn were susceptible to cold during winter, but neither cold nor warm weather appeared major determinants at other times of the year. Non-Sami, on the other hand, were mainly affected by weather in summer and autumn. This suggests that Sami, who over centuries had adapted to the harsh Nordic climate, could cope fairly well with temperature variations, except for the very low extremes during winter, when risk of perinatal death was already high. The observed ethnic heterogeneity in vulnerability to temperature extremes most likely reflect the Sami’s and non-Sami’s specific working and living conditions. Children’s vulnerability to seasonality and environmental factors, including weather extremes, depends on socio-economic status, living conditions and behavioural patterns of parents. In Wales and England, seasonality of stillbirth risk and neonatal mortality decreased substantially during the 20^th^ century, supposedly owing to the introduction of central heating [44]. This is in line with our findings that Sami, who had poorer housing conditions, rather than non-Sami infants, were affected by cold extremes during winter. Thus, ethnicity showed to be a strong effect modifier of the weather-mortality relationship. Other factors such as socio-economic status and economic development were not investigated in this study, but might as well cause effect modification within each ethnic group. The study by Ekamper et al. found a declining effect of weather extremes on infant mortality in the course of socio-economic development [41]. Contemporary studies from California, US, and from Uganda indicated ethnic heterogeneity in heat effects on birth outcomes [21, 45]. These findings across time and space demonstrate how environmental and social contexts contribute to climate vulnerability in resource-poor communities.

### Strengths and limitations

The present study accounted for ethnicity, seasonality and non-linearity in the relationship between temperature and perinatal mortality. For this health outcome, there is still a lack of studies with historical or contemporary populations, least in higher latitude climates such as the Subarctic. We had the opportunity to use a unique population sample including vital data, information of ethnicity and date and place of birth and death. We also accessed early temperature measurements prior 1860, when more regular recordings became available. These measurements have however some limitations, mainly the monthly scale of available temperature data, and the distance between weather stations and study locations. While monthly data do not allow for the analysis of associations between daily temperature and risk of stillbirth or death in the first week of live, they can provide us with a general understanding of short-term weather impacts on perinatal mortality. Using rather distant weather measurements and–in the case of Umeå –reconstructed temperature data [34] introduced–albeit not a bias–imprecision in effect estimates, limiting the certainty of conclusions. However, our main exposure variable was categorized into warm or cold extremes, which are less sensitive to spatial variations–we can assume that these reflected rather well month-to-month variations of extreme weather at the study locations, and that results are sufficiently robust.

Birth and death dates might not always have been quite correct at that time, in particular for the semi-nomad Sami, so analyses at the monthly level can partly avoid this limitation, although it might also cause misclassification of perinatal deaths. Even today, there is a large degree of underreporting of stillbirths and early neonatal deaths in many countries [9], and most certainly was also present in our pre-industrial, remote Sápmi population. Spatial and temporal variations of underreporting might have introduced a bias when assessing patterns of perinatal mortality rates.

## Conclusions

Our study in 19^th^ century Sweden showed that the association of high and low temperature with perinatal mortality varied both by ethnicity and by season. Reindeer herding Sami appeared to be vulnerable mainly to cold in winter, and peasant non-Sami settlers to weather extremes in summer and autumn. It also highlighted the non-linearity of the temperature-mortality relationship, as has been shown for all-age mortality in a large number of contemporary studies.

This study demonstrated that even in a subarctic climate, cold-related mortality risk might be low, while high temperature might be protective only among certain vulnerable groups. Living conditions influence physiological vulnerability to temperature extremes, while culture and lifestyles determine the degree of exposure to heat and cold. Promoting awareness of these interrelated determinants of health provides opportunities for prevention. Primary health care providers, including midwives, but also community leaders and families need to be informed about risks related to extreme temperatures for the unborn and newborn, and about means of prevention, such as protection from cold and heat exposures. Most importantly, health care providers need to consider the cultural and socio-economic context that shapes vulnerabilities of specific population groups such as indigenous people and ethnic minorities.

## Supporting information

S1 Table Perinatal mortality by temperature extremes with co-variables. **Odds ratios with 95% confidence intervals**.
Cold lag 0: Temperature <10^th^ percentile in month of birth, warm lag 0: Temperature >90^th^ percentile in month of birth. Cold lag 1, warm lag 1: cold resp. warm in month before birth. Reference: moderate temperature (10^th^ to 90^th^ percentile). Period: year of birth. Reference: 1860–1895. Model 1: Cold and warm at lag 0 only. Model 2: Cold and warm at lag 0 and at lag 1. Model 3: Cold and warm at lag 0 and at lag 1, and period of birth. Model 4: Cold and warm at lag 0 and at lag 1, period of birth, region and sex. All values are odds ratios with 95% confidence intervals.
(DOCX)

S2 Table Sensitivity analyses, north Sápmi data only. **Model 3: Perinatal mortality by temperature extremes at lag 0 and lag 1, and by period. Odds ratios with 95% confidence intervals**.
Cold lag 0: Temperature <10^th^ percentile in month of birth, warm lag 0: Temperature >90^th^ percentile in month of birth. Cold lag 1, warm lag 1: cold resp. warm in month before birth. Reference: moderate temperature (10^th^ to 90^th^ percentile). Period: year of birth. Reference: 1860–1895.
South Sápmi data (population only from southern parishes, observed and extrapolated temperature data from Umeå) were excluded in these analyses due to the less precise temperature data.
(DOCX)
