# Supplementary material for: Weather extremes and perinatal mortality – Seasonal and ethnic differences in northern Sweden, 1800-1895
Source: PLoS One. 2019 Oct 22;14(10):e0223538. doi: 10.1371/journal.pone.0223538 (PMC6804957; doi:10.1371/journal.pone.0223538)
Supplement: S1 Table — Odds ratios with 95% confidence intervals. Cold lag 0: Temperature <10th percentile in month of birth, warm lag 0: Temperature >90th percentile in month of birth. Cold lag 1, warm lag 1: cold resp. warm in month before birth. Reference: moderate temperature (10th to 90th percentile). Period: year of birth. Reference: 1860–1895. Model 1: Cold and warm at lag 0 only. Model 2: Cold and warm at lag 0 and at lag 1. Model 3: Cold and warm at lag 0 and at lag 1, and period of birth. Model 4: Cold and warm at lag 0 and at lag 1, period of birth, region and sex. All values are odds ratios with 95% confidence intervals. (DOCX) [file pone.0223538.s001.docx]

**S1 Table: Perinatal mortality by temperature extremes and co-variables. Odds ratios with 95% confidence intervals**

| **Winter** | **Sami** | | | | **Non-Sami** | | | |
| --- | --- | --- | --- | --- | --- | --- | --- | --- |
|  | **Model 1** | **Model 2** | **Model 3** | **Model 4** | **Model 1** | **Model 2** | **Model 3** | **Model 4** |
| **Cold lag 0** | **1.85** (1.22-2.80) | **1.86** (1.22-2.82) | **1.91** (1.26-2.92) | **1.91** (1.26-2.92) | **1.10** (0.62-1.93) | **1.08** (0.61-1.90) | **1.15** (0.65-2.02) | **1.17** (0.66-2.07) |
| **Warm lag 0** | **0.90** (0.54-1.50) | **0.88** (0.52-1.50) | **0.86** (0.51-1.47) | **0.87** (0.51-1.49) | **0.67** (0.37-1.19) | **0.74** (0.41-1.34) | **0.73** (0.40-1.33) | **0.73 (**0.40-1.33) |
| **Cold lag 1** | - | **1.06** (0.64-1.76) | **1.05** (0.63-1.74) | **1.07** (0.65-1.79) | - | **1.22** (0.72-2.05) | **1.26** (0.75-2.13) | **1.29** (0.76-2.18) |
| **Warm lag 1** | - | **1.09** (0.67-1.80) | **1.11** (0.68-1.83) | **1.09** (0.66-1.80) | - | **0.69** (0.37-1.28) | **0.68** (0.37-1.27) | **0.68** (0.37-1.27) |
| **Period 1800-1829** | - | - | **1.27** (0.90-1.79) | **1.25** (0.88-1.76) | - | - | **0.83** (0.47-1.47) | **0.84** (0.48-1.49) |
| **Period 1830-1859** | - | - | **0.54** (0.36-0.81) | **0.54** (0.36-0.81) | - | - | **0.64** (0.41-0.99) | **0.64** (0.41-1.00) |
| **Region north** | - | - | **-** | **1.47** (0.86-2.52) | - | - | - | **1.26** (0.89-1.79) |
| **Sex female** | - | - | **-** | **0.68** (0.50-0.92) | - | - | - | **0.80** (0.58-1.10) |
|  | | | | | | | | |
| **Spring** | **Sami** | | | | **Non-Sami** | | | |
|  | **Model 1** | **Model 2** | **Model 3** | **Model 4** | **Model 1** | **Model 2** | **Model 3** | **Model 4** |
| **Cold lag 0** | **0.91** (0.53-1.58) | **0.87** (0.50-1.52) | **0.84** (0.48-1.46) | **0.84** (0.48-1.47) | **1.09** (0.66-1.78) | **1.24** (0.75-2.06) | **1.21** (0.73-2.00) | **1.24** (0.75-2.06) |
| **Warm lag 0** | **0.89** (0.49-1.63) | **0.92** (0.50-1.69) | **0.90** (0.49-1.67) | **0.90** (0.49-1.66) | **1.00** (0.54-1.88) | **1.03** (0.55-1.93) | **1.01** (0.53-1.91) | **1.00** (0.53-1.90) |
| **Cold lag 1** | - | **1.21** (0.73-1.99) | **1.20** (0.73-1.99) | **1.23** (0.74-2.03) | - | **0.40** (0.19-0.84) | **0.41** (0.19-0.85) | **0.40** (0.19-0.84) |
| **Warm lag 1** | - | **0.84** (0.46-1.55) | **0.85** (0.46-1.57) | **0.86** (0.47-1.59) | - | **0.79** (0.44-1.41) | **0.77** (0.43-1.38) | **0.78** (0.44-1.40) |
| **Period 1800-1829** | - | - | **1.25** (0.83-1.88) | **1.21** (0.81-1.83) | - | - | **1.35** (0.83-2.21) | **1.44** (0.88-2.35) |
| **Period 1830-1859** | - | - | **0.80** (0.54-1.19) | **0.79** (0.54-1.18) | - | - | **0.75** (0.48-1.17) | **0.78** (0.50-1.22) |
| **Region north** | - | - | **-** | **1.34** (0.80-2.24) | - | - |  | **1.35** (0.96-1.92) |
| **Sex female** | - | - | **-** | **0.69** (0.50-0.95) | - | - |  | **0.90** (0.66-1.24) |
|  | | | | | | | | |
| **Summer** | **Sami** | | | | **Non-Sami** | | | |
|  | **Model 1** | **Model 2** | **Model 3** | **Model 4** | **Model 1** | **Model 2** | **Model 3** | **Model 4** |
| **Cold lag 0** | **0.74** (0.39-1.39) | **0.7**1 (0.38-1.35) | **0.73** (0.39-1.38) | **0.73** (0.39-1.38) | **1.27** (0.78-2.06) | **1.37** (0.84-2.25) | **1.37** (0.84-2.24) | **1.40** (0.86-2.30) |
| **Warm lag 0** | **1.11** (0.63-1.98) | **1.19** (0.67-2.12) | **1.24** (0.69-2.22) | **1.26** (0.70-2.27) | **0.18** (0.04-0.73) | **0.16** (0.04-0.66) | **0.20** (0.05-0.81) | **0.20** (0.05-0.84) |
| **Cold lag 1** | - | **0.97** (0.54-1.72) | **0.87** (0.49-1.55) | **0.86** (0.48-1.54) | - | **0.54** (0.28-1.05) | **0.49** (0.25-0.95) | **0.49** (0.26-0.96) |
| **Warm lag 1** | - | **0.42** (0.17-1.05) | **0.43** (0.17-1.09) | **0.44** (0.18-1.10) | - | **1.43** (0.81-2.55) | **1.65** (0.92-2.97) | **1.61** (0.90-2.89) |
| **Period 1800-1829** | - | - | **1.00** (0.63-1.58) | **1.00** (0.63-1.58) | - | - | **0.54** (0.28-1.05) | **0.60** (0.31-1.18) |
| **Period 1830-1859** | - | - | **0.53** (0.33-0.85) | **0.54** (0.34-0.86) | - | - | **0.39** (0.22-0.70) | **0.41** (0.23-0.74) |
| **Region north** | - | - | **-** | **1.17** (0.69-1.98) | - | - |  | **1.72** (1.16-2.56) |
| **Sex female** | - | - | **-** | **0.75** (0.52-1.07) | - | - |  | **0.95** (0.67-1.33) |
|  | | | | | | | | |
| **Autumn** | **Sami** | | | | **Non-Sami** | | | |
|  | **Model 1** | **Model 2** | **Model 3** | **Model 4** | **Model 1** | **Model 2** | **Model 3** | **Model 4** |
| **Cold lag 0** | **1.00** (0.61-1.63) | **0.99** (0.60-1.62) | **0.96** (0.58-1.57) | **0.96** (0.58-1.57) | **0.46** (0.22-0.94) | **0.42** (0.20-0.88) | **0.39** (0.19-0.82) | **0.40** (0.19-0.83) |
| **Warm lag 0** | **0.86** (0.50-1.49) | **0.84** (0.49-1.45) | **0.88** (0.51-1.52) | **0.87** (0.50-1.52) | **1.17** (0.70-1.95) | **1.18** (0.71-1.98) | **1.20** (0.72-2.01) | **1.18** (0.71-1.99) |
| **Cold lag 1** | - | **0.88** (0.53-1.46) | **0.86** (0.52-1.43) | **0.86** (0.52-1.42) | - | **1.31** (0.79-2.17) | **1.27** (0.76-2.11) | **1.29** (0.78-2.15) |
| **Warm lag 1** | - | **0.72** (0.39-1.31) | **0.76** (0.41-1.39) | **0.76** (0.41-1.39) | - | **0.72** (0.35-1.50) | **0.80** (0.38-1.67) | **0.78** (0.38-1.64) |
| **Period 1800-1829** | - | - | **1.65** (1.18-2.32) | **1.62** (1.15-2.28) | - | - | **0.90** (0.50-1.63) | **0.96** (0.53-1.73) |
| **Period 1830-1859** | - | - | **0.57** (0.38-0.87) | **0.57** (0.38-0.86) | - | - | **0.37** (0.21-0.67) | **0.39** (0.22-0.70) |
| **Region north** | - | - | **-** | **1.35** (0.81-2.25) | - | - |  | **1.77** (1.18-2.65) |
| **Sex female** | - | - | **-** | **0.94** (0.69-1.27) | - | - |  | **0.84** (0.60-1.18) |

Cold lag 0: Temperature <10^th^ percentile in month of birth, warm lag 0: Temperature >90^th^ percentile in month of birth. Cold lag 1, warm lag 1: cold resp. warm in month before birth. Reference: moderate temperature (10^th^ to 90^th^ percentile). Period: year of birth. Reference: 1860-1895. Region north: place of birth, north Sápmi. Reference: south Sápmi. Sex female: reference males.

Model 1: Cold and warm at lag 0 only. Model 2: Cold and warm at lag 0 and at lag 1. Model 3: Cold and warm at lag 0 and at lag 1, and period of birth. Model 4: Cold and warm at lag 0 and at lag 1, by period of birth, region and sex.

All values are odds ratios with 95% confidence intervals.
