# Supplementary material for: Weather extremes and perinatal mortality – Seasonal and ethnic differences in northern Sweden, 1800-1895
Source: PLoS One. 2019 Oct 22;14(10):e0223538. doi: 10.1371/journal.pone.0223538 (PMC6804957; doi:10.1371/journal.pone.0223538)
Supplement: S2 Table — Model 3: Perinatal mortality by temperature extremes at lag 0 and lag 1, and by period. Odds ratios with 95% confidence intervals. Cold lag 0: Temperature <10th percentile in month of birth, warm lag 0: Temperature >90th percentile in month of birth. Cold lag 1, warm lag 1: cold resp. warm in month before birth. Reference: moderate temperature (10th to 90th percentile). Period: year of birth. Reference: 1860–1895. South Sápmi data (population only from southern parishes, observed and extrapolated temperature data from Umeå) were excluded in these analyses due to the less precise temperature data. (DOCX) [file pone.0223538.s002.docx]

**S2 Table: Sensitivity analyses, north Sápmi data only. Model 3: Perinatal mortality by temperature extremes at lag 0 and lag 1, and by period. Odds ratios with 95% confidence intervals.**

|  | **WINTER** | | **SPRING** | | **SUMMER** | | **AUTUMN** | |
| --- | --- | --- | --- | --- | --- | --- | --- | --- |
|  | **Sami** | **Non-Sami** | **Sami** | **Non-Sami** | **Sami** | **Non-Sami** | **Sami** | **Non-Sami** |
| **Cold lag 0** | **1.74** (1.11-2.73) | **1.61** (0.84-3.11) | **0.95** (0.53-1.70) | **1.39** (0.73-2.61) | **0.69** (0.34-1.40) | **1.45** (0.80-2.63) | **0.99** (0.59-1.67) | **0.38** (0.16-0.89) |
| **Warm lag 0** | **0.92** (0.54-1.57) | **1.01** (0.53-1.93) | **0.97** (0.52-1.81) | **1.24** (0.60-2.54) | **1.16** (0.60-2.23) | **0.30** (0.07-1.23) | **0.96** (0.55-1.67) | **1.00** (0.55-1.84) |
| **Cold lag 1** | **1.02** (0.60-1.76) | **1.17** (0.59-2.31) | **1.18** (0.69-2.02) | **0.21** (0.06-0.67) | **1.06** (0.59-1.91) | **0.40** (0.17-0.93) | **0.78** (0.45-1.35) | **1.36** (0.77-2.42) |
| **Warm lag 1** | **1.16** (0.70-1.93) | **0.65** (0.31-1.34) | **0.84** (0.44-1.59) | **0.83** (0.41-1.68) | **0.56** (0.22-1.40) | **1.98** (1.07-3.67) | **0.85** (0.46-1.56) | **0.71** (0.30-1.66) |
| **Period 1800-1829** | **1.21** (0.84-1.74) | **1.14** (0.59-2.20) | **1.36** (0.89-2.08) | **1.60** (0.86-2.97) | **0.96** (0.59-1.56) | **0.38** (0.14-1.05) | **1.59** (1.11-2.28) | **0.85** (0.40-1.79) |
| **Period 1830-1859** | **0.57** (0.38-0.87) | **0.68** (0.40-1.18) | **0.90** (0.59-1.36) | **0.69** (0.39-1.23) | **0.57** (0.35-0.95) | **0.49** (0.26-0.94) | **0.59** (0.38-0.90) | **0.55** (0.31-1.00) |

Cold lag 0: Temperature <10^th^ percentile in month of birth, warm lag 0: Temperature >90^th^ percentile in month of birth. Cold lag 1, warm lag 1: cold resp. warm in month before birth. Reference: moderate temperature (10^th^ to 90^th^ percentile). Period: year of birth. Reference: 1860-1895.

South Sápmi data (population only from southern parishes, observed and extrapolated temperature data from Umeå) were excluded in these analyses due to the less precise temperature data.
